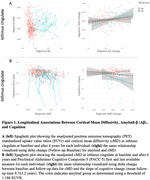# Longitudinal associations of microstructural changes, amyloid, and cognition in preclinical Alzheimer's disease

**DOI:** 10.1002/alz.089927

**Published:** 2025-01-09

**Authors:** Patrizia Vannini, Giacomo Bertazzoli, Elena Rodriguez‐Vieitez, Max Ramseyer, Ibai Diez, Jorge Sepulcre, Bernard J Hanseeuw, Cristina Lois, Geoffroy Pierre Gagliardi, Carl‐Fredrik Westin, Keith A Johnson, Reisa A Sperling, Gad A Marshall

**Affiliations:** ^1^ Massachusetts General Hospital, Boston, MA USA; ^2^ Brigham and Women's Hospital, Boston, MA USA; ^3^ Harvard Medical School, Boston, MA USA; ^4^ Division of Neurogeriatrics, Center for Alzheimer Research, Department of Neurobiology, Care Sciences and Society, Karolinska Institutet, Stockholm Sweden; ^5^ Athinoula A. Martinos Center for Biomedical Imaging, Charlestown, MA USA; ^6^ Saint Luc University Hospital, Université Catholique de Louvain, Brussels Belgium

## Abstract

**Background:**

A novel method using diffusion‐weighted imaging (DWI) allows for assessing microstructural injury in the gray matter by measuring cortical mean diffusivity (cMD). Previous studies have shown that altered cMD is related to amyloid (Aβ) cross‐sectionally and can predict longitudinal cognitive decline and clinical progression, suggesting utility in clinical trials. However, the longitudinal associations between these measures are unknown. This study aimed to investigate the longitudinal associations among cMD, Aβ, and cognition, in individuals who were cognitively normal (CN) at baseline.

**Methods:**

The study sample comprised n=156 CN older adults (73.8±6.1 y/o;92 [59%] women) from the Harvard Aging Brain Study. All participants underwent structural MRI, DWI, and 11C‐Pittsburgh compound‐B‐PET at baseline and after 4 years. Cognitive assessment using the Preclinical Alzheimer Cognitive Composite‐5 (PACC‐5) was collected at baseline and yearly for an average of 9.7±3.2 years. Generalized linear models (GLMs) were used to assess the regional (focusing on brain areas vulnerable to early Aβ accumulation) change in Aβ on change in cMD, covarying for baseline cortical thickness, change in cortical thickness, age, and sex. GLMs were used to assess the impact of change in regional cMD by Aβ group, on the rate of change in cognition covarying for baseline cMD, baseline age, sex, and education.

**Results:**

Overall, Aβ and cMD signal increased over the 4‐year period. 44 participants (28%) exhibited an initial high Aβ burden (Aβ+, thresholded using 1.186 SUVR). An increase in Aβ pathology (over 4 years) predicted a concomitant increase in cMD in the isthmus cingulate (ßstd=6.8e‐06;p=0.019). Post hoc stratified analyses by Aβ‐status revealed that this effect was only observed in the Aβ+individuals (ßstd=1.6e‐05;p=0.023, Figure 1A). Moreover, a significant interaction between Aβ and cMD signal predicted cognition, such that an antecedent increase in cMD was related to a steeper cognitive decline in Aβ+ individuals (ßstd=‐0.061;p=0.004, Figure 1B).

**Conclusion:**

Amyloid accumulation is associated with a concomitant increase in cMD, suggesting that it may reflect microstructural alterations in response to Aβ aggregation. Moreover, an increase in cMD over time predicted cognitive decline in at‐risk individuals. Overall, metrics of cortical diffusion may provide important outcome measures in Aβ‐modifying clinical trials for detecting drug‐induced changes in cortical microstructure.